# Supplementary material for: Clinical Impact of Implementing a Nurse-Led Adverse Drug Reaction Profile in Older Adults Prescribed Multiple Medicines in UK Primary Care: A Study Protocol for a Cluster-Randomised Controlled Trial
Source: Pharmacy (Basel). 2022 Apr 28;10(3):52. doi: 10.3390/pharmacy10030052 (PMC9149816; doi:10.3390/pharmacy10030052)
Supplement: Supplementary file 1 [file pharmacy-10-00052-s001.zip › pharmacy-1644687-supplementary/Supplementary Figure S1_The logic model.pdf]

Supplementary figure 1 (Figure S1): The logic model of ADRe intervention

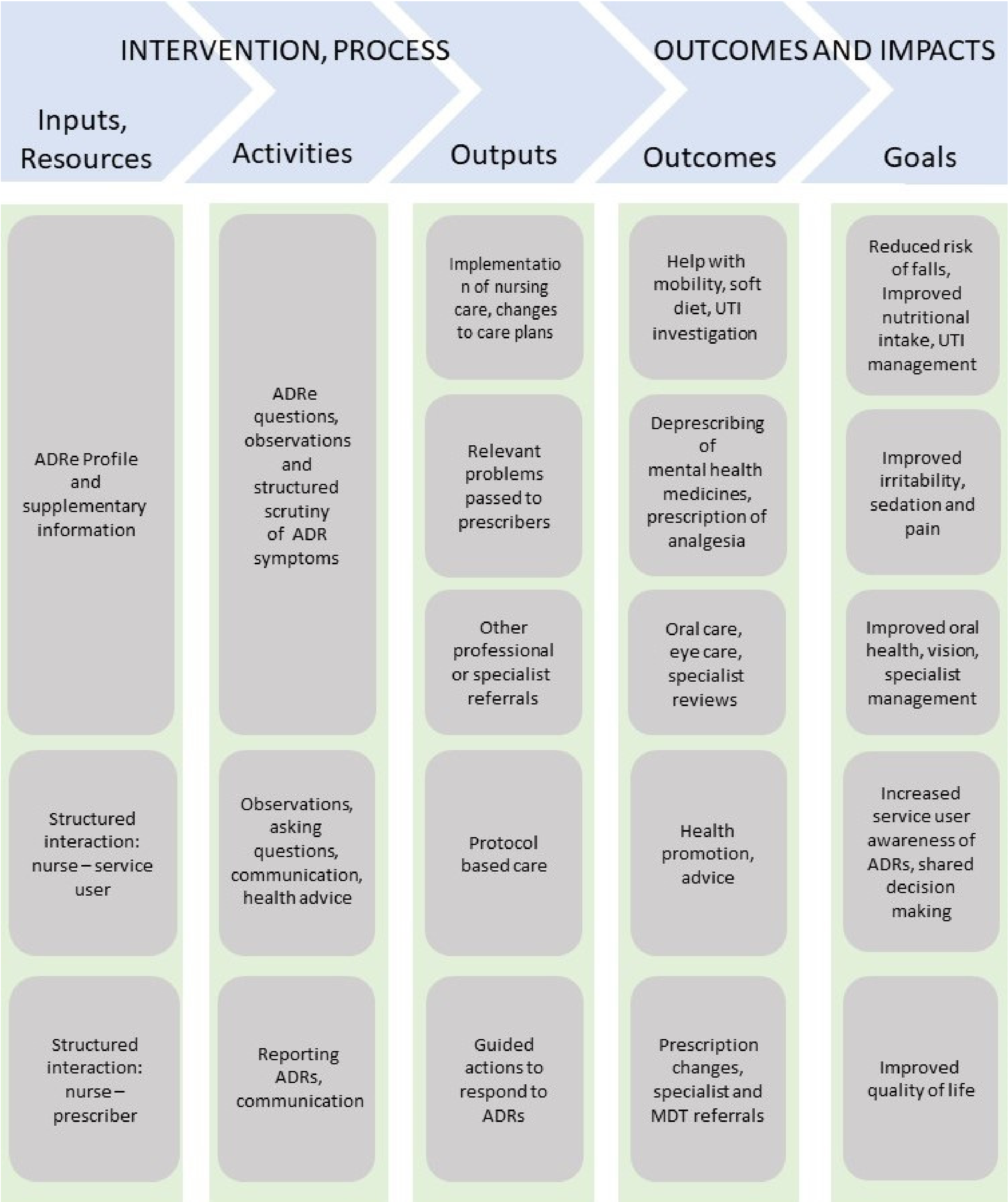

Notes: Model based on W.K. Kellogg Foundation Logic Model Development Guide (2004), and Knowlton (2012). Adapted from Jordan et al. (2018).
