# Supplementary material for: Clinical Impact of Implementing a Nurse-Led Adverse Drug Reaction Profile in Older Adults Prescribed Multiple Medicines in UK Primary Care: A Study Protocol for a Cluster-Randomised Controlled Trial
Source: Pharmacy (Basel). 2022 Apr 28;10(3):52. doi: 10.3390/pharmacy10030052 (PMC9149816; doi:10.3390/pharmacy10030052)
Supplement: Supplementary file 1 [file pharmacy-10-00052-s001.zip › pharmacy-1644687-supplementary/Supplementary File S1_Safety reporting.pdf]

Supplementary file 1 (File S1): Safety Reporting

Overview of the Safety Reporting Process/Pharmacoviligance responsibilities

It is extremely unlikely that the implementation of the ADRe Profile (checklist) should cause any harm. There were no reported safety issues in previous studies (Jordan, 2002; Gabe et al., 2014; Jordan et al., 2015; Jones et al., 2016; Jordan et al., 2019). It is unlikely that administration of ADRe per se would lead to harm: actions emanating from use of ADRe (for example, deprescribing) have potential for harm, but these are the responsibility of the attending clinicians.

The ADRe Profile has been developed to identify and support amelioration of potential side effects from commonly prescribed medicines. As part of the study protocol, the completed ADRe Profile and the list of patient's medicines is reviewed by the study pharmacist and, if needed, the patient's GP. Any untoward events will be reported to the study steering group, which comprises a consultant pharmacist, a pharmacy lecturer and two service users.

General Definitions

Adverse Event (AE)

An AE is any untoward medical occurrence in a subject to whom a medicinal product has been administered, including occurrences which are not necessarily caused by or related to that product. An AE can therefore be any unfavourable and unintended sign (including an abnormal laboratory finding), symptom, or disease temporally associated with the use of a medicinal (investigational) product, whether or not related to the medicinal product. symptom or disease temporarily associated with study activities (International Conference on Harmonisation [ICH], 1996).

Serious Adverse Event (SAE)

Serious ADRs are those which result in death, life-threatening conditions, persistent or significant disability or incapacity, hospitalization, prolonged hospitalization, congenital anomalies (ICH, 1996).

An SAE fulfils at least one of the following criteria:

· Is fatal – results in death (NOTE: death is an outcome, not an event)

· Is life-threatening

· Requires inpatient hospitalisation or prolongation of existing hospitalisation

· Results in persistent or significant disability/incapacity

· Is a congenital anomaly/birth defect

· Is otherwise considered medically significant by the Investigator

- **Adverse Event (AE)**

If an adverse event is identified or reported, it will be recorded in the participant's medical records. Any adverse events will be assessed by the clinical team and the chief investigator. Any adverse events arising as a result of the ADRe checklist will be passed to the sponsor's representative and the participant's responsible clinician for assessment. No adverse events have arisen as a result of using the ADRe checklists in 2 trials and 3 observation studies. Previous research has not identified any harm resulting from completing the ADRe Profile checklist. We are not planning on establishing a Data Monitoring Committee.

- **Serious Adverse Event (SAE)**

Previous research has not identified any harm resulting from completing the ADRe Profile checklist. We are not planning on establishing a Data Monitoring Committee.

Investigators' Assessment

- Seriousness

The Chief/Principal Investigator or the clinical team responsible for the care of the patient will assess whether the event is serious.

- Causality

The Chief Investigator will be responsible for assessing the causality.

- Expectedness

It is unlikely that completing a checklist should cause any serious adverse events.

- Severity

The Chief Investigator will assess the severity of the event according to the following definitions:

Mild: Some discomfort noted but without disruption of daily life

Moderate: Discomfort enough to affect/reduce normal activity

Severe: Complete inability to perform daily activities and lead a normal life

Notification and reporting Adverse Events or Reactions

Any Adverse Event will be recorded in the study file and the participant will be followed up the research team. The Adverse Event will be documented in the participant's medical notes and the CRF.

Notification and Reporting of Serious Adverse Events

Related and unexpected Serious Adverse Events will be reported to the sponsor within 24 hours of learning of the event and to the Main REC within 15 days.

Urgent Safety Measures

Should the Chief Investigator need to take any urgent safety measures to ensure the safety and protection of the trial participants from any immediate hazard to their health and safety, any such measures will be taken immediately. The Sponsor and the Main Ethics Committee will be informed immediately via telephone and in writing within 3 days (in a form of a substantial amendment). The Sponsor will be sent a copy of all correspondence.

Annual Safety Reporting

The Chief Investigator will send the Annual Progress Report to the main REC (using NRES template).

Procedures for reporting blinded ' unexpected' and related' SAEs

N/A

References:

Gabe, Marie E, Murphy, Fiona, Davies, Gwyneth A, Russell, Ian T, & Jordan, Susan. (2014). Medication monitoring in a nurse-led respiratory outpatient clinic: pragmatic randomised trial of the West Wales Adverse Drug Reaction Profile. PloS One, 9(5), e96682–e96682. https://doi.org/10.1371/journal.pone.0096682

International Conference on Harmonisation (ICH). (1996). *ICH Harmonised Tripartite Guideline for Good Clinical Practice*. Institute of Clinical Research, Marlow, Buckinghamshire. 1996. Available at: http://www.ich.org/fileadmin/Public_Web_Site/ICH_Products/Guidelines/Efficacy/E6/E6_R1_Guideline.pdf (Accessed January 31, 2018).

Jones, Richard, Moyle, Christopher, & Jordan, Sue. (2016). Nurse-led medicines monitoring: a study examining the effects of the West Wales Adverse Drug Reaction Profile. Nursing Standard, 31(14), 42–53. https://doi.org/10.7748/ns.2016.e10447

Jordan, Sue, Tunnicliffe, Carol, & Sykes, Alan. (2002). Minimizing side-effects: the clinical impact of nurse-administered 'side-effect' checklists. Journal of Advanced Nursing, 37(2), 155–165. https://doi.org/10.1046/j.1365-2648.2002.02064.x

Jordan, Susan, Gabe-Walters, Marie Ellenor, Watkins, Alan, Humphreys, Ioan, Newson, Louise, Snelgrove, Sherrill, & Dennis, Michael S. (2015). Nurse-Led Medicines' Monitoring for Patients with Dementia in Care Homes: A Pragmatic Cohort Stepped Wedge Cluster Randomised Trial. PloS One, 10(10), e0140203–e0140203. https://doi.org/10.1371/journal.pone.0140203

Jordan, Sue, Banner, Timothy, Gabe-Walters, Marie, Mikhail, Jane M, Panes, Gerwyn, Round, Jeff, Snelgrove, Sherrill, Storey, Mel, & Hughes, David. (2019). Nurse-led medicines' monitoring in care homes, implementing the Adverse Drug Reaction (ADRe) Profile improvement initiative for mental health medicines: An observational and interview study. PloS One, 14(9), e0220885–e0220885. https://doi.org/10.1371/journal.pone.0220885

An SAE/SUSAR reporting Organogram

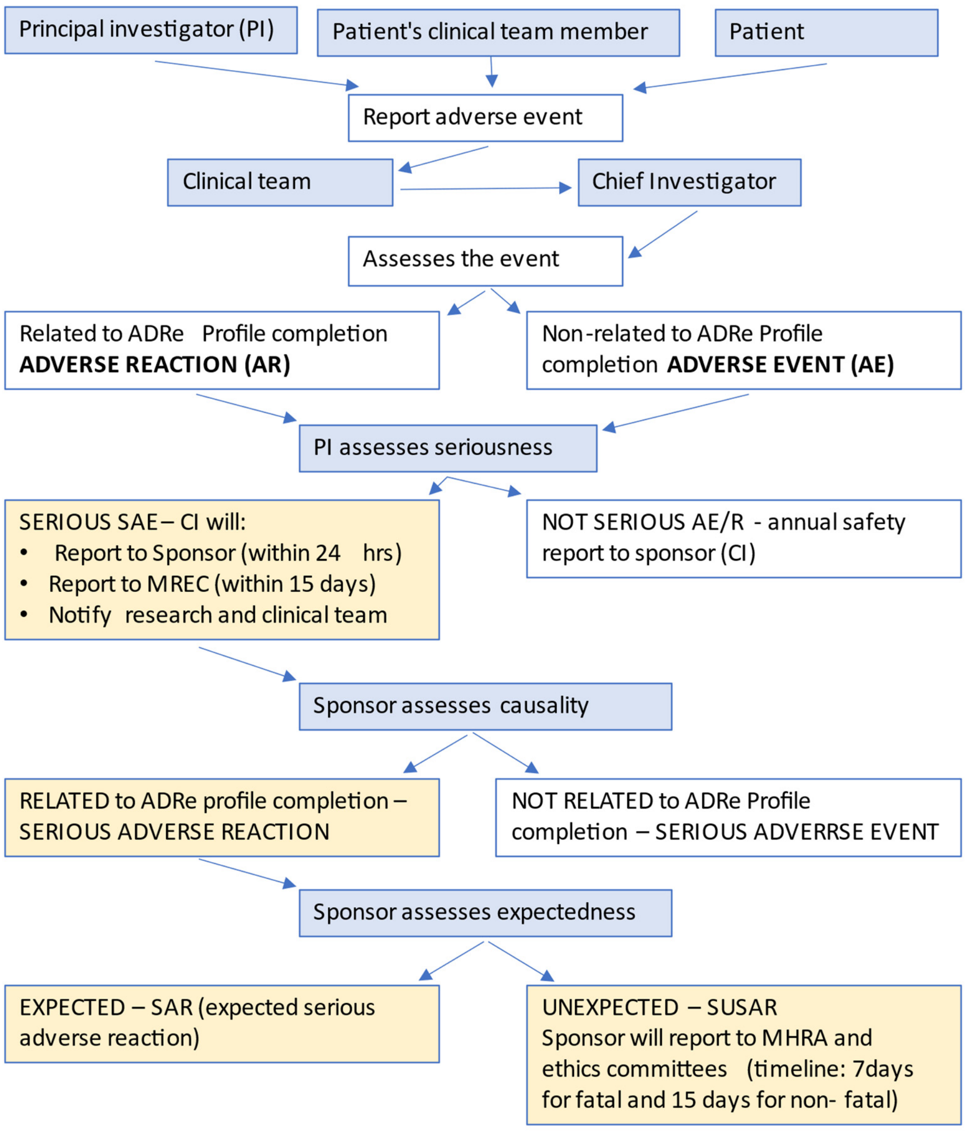

PI in liaison with academic supervisory team will act as a back -up for CI

Notes: PI – principal investigator, CI – chief investigator
