# Supplementary material for: Clinical Impact of Implementing a Nurse-Led Adverse Drug Reaction Profile in Older Adults Prescribed Multiple Medicines in UK Primary Care: A Study Protocol for a Cluster-Randomised Controlled Trial
Source: Pharmacy (Basel). 2022 Apr 28;10(3):52. doi: 10.3390/pharmacy10030052 (PMC9149816; doi:10.3390/pharmacy10030052)
Supplement: Supplementary file 1 [file pharmacy-10-00052-s001.zip › pharmacy-1644687-supplementary/Supplementary File S2_Survey questions.pdf]

Supplementary file 2 (File S2): Survey questions

Primary outcome measures relating to the survey

1) Description of stakeholder views on the **effectiveness of implementing the ADRe Profile** (survey rating of the ADRe Profile - Likert scale). A brief survey will be distributed to the main stakeholders (patients, nurses, GP's and pharmacists) following completion of Randomised Controlled Trial
2) Description of stakeholder views on ADRe **Profile implementation feasibility** (eliciting interview themes). Semi-structured interview with a purposive sample of the main stakeholders (patients, nurses, GP's and pharmacists) to explore their views on feasibility of ADRe Profile integration in GP practices.

Secondary outcome measures relating to Interviews/survey

3) Description of stakeholders' views on **multidisciplinary collaboration** (eliciting interview themes). Semi-structured interviews with the main stakeholders (patients, nurses, GP's and pharmacists) will explore their views on whether and how the ADRe Profile delivered clinical benefits and / or contributed to multidisciplinary collaboration between nurses, doctors and pharmacists.
4) Description of the patients' views on the **contribution of ADRe Profile to their health and well-being and patient-centred care** (eliciting interview themes). Semi-structured interviews with the patients will seek their views on whether and how ADRe Profile contributes to their health and well-being and patient-centred care. [Time Frame: 22 months from the start of the study]
5) **Time spent** completing one ADRe Profile

Survey for patients:

In your opinion, does the ADRe Profile benefit people who take multiple medicines? (1)

No	Not yet but maybe in future	Yes	Don’t know

Do you think you benefited from using the ADRe Profile? (1)

No	Not yet but maybe in future	Yes	Don’t know

If you benefited, how important was the benefit to you? (1)

Not important	Slightly important	Very important	Don’t know

Possible probe question: Please describe any benefits you feel you received.

How easy/difficult did you find completing the ADRe Profile independently? (2)

Difficult	Relatively easy	Easy	Don't know

Possible probe question: Please tell us what was difficult and what was easy. This will help us improve in the future.

How long did you spend completing the ADRe Profile independently? (5)

15 mins	30 mins	45 mins	1 hour or longer

How easy/difficult did you find completing the ADRe Profile with the nurse/researcher? (2)

Difficult	Relatively easy	Easy	Don't know

Possible probe question: Please tell us what was difficult and what was easy. This will help us improve in the future.

How long did it take to complete the ADRe Profile with the nurse/researcher? (5)

15 mins	30 mins	45 mins	1 hour or longer

Would you be willing/able to complete the ADRe Profile regularly (4 times per year) to contribute to your medications review? (2)

No	Yes, but less often	Yes	Don't know

How would you rate the health nurses', doctors' and pharmacists' collaboration during the ADRe project? (3)

Poor	Good	Excellent	Don't know

Possible probe question: Please tell us why you gave this rating.

Was your unique experience taken into account, did you feel you were you listened to? (4)

No	Yes, to some extent	Yes, a lot	Don't know

Possible probe question: Please tell us why you gave this rating.

Did you feel involved in planning and decisions made about your care when using the ADRe Profile? (4)

No	Yes, to some extent	Yes, a lot	Don't know

Possible probe question: Please tell us why you gave this rating.

Is there anything else you would like us to know?

Thank you for your time in completing this survey.
