# Supplementary material for: Clinical Impact of Implementing a Nurse-Led Adverse Drug Reaction Profile in Older Adults Prescribed Multiple Medicines in UK Primary Care: A Study Protocol for a Cluster-Randomised Controlled Trial
Source: Pharmacy (Basel). 2022 Apr 28;10(3):52. doi: 10.3390/pharmacy10030052 (PMC9149816; doi:10.3390/pharmacy10030052)
Supplement: Supplementary file 1 [file pharmacy-10-00052-s001.zip › pharmacy-1644687-supplementary/Supplementary File S3_Interview guide.pdf]

Supplemetary file 3 (File S3): Interview guides

Interview guide for patient participants

Interviews aim to understand:

1. ADRe effectiveness
2. Feasibility of implementation
3. Views on multidisciplinary collaboration
4. Contribution of ADRe Profile to patient-centred care

Exploration of views on ADRe Profile effectiveness

- Did you notice any benefit to you from the use of ADRe Profile?
    - If YES to the previous question: How much do you value the benefit the ADRe intervention brought you?
    - Why/how did the benefit occur?
- In your opinion, why does ADRe Profile work/ or not work?
- Has participation in the study changed your thinking about medicines and their side effects? In what way?
- Would you like to see the ADRe Profile continue to be used? Why?

Exploration of the feasibility of routine implementation

- Before you used ADRe, did your prescriber speak to you about your medicines? (How? How often? How long did the conversation last? Was it useful? Did you have an opportunity to express your views/problems/concerns?)
    - If YES to the previous question: How does using ADRe compare with usual way of keeping an eye on what medicines are prescribed for you? (Is it different? /How is it different?)
- How would you describe the purpose of the ADRe Profile? /What is the point of using ADRe? /Is ADRe a good idea?
- Was the time you spent completing ADRe spent usefully? /Would you be happy to complete the ADRe Profile every 3-6 months?
- Did you encounter any problems or difficulties in using ADRe?
    - If YES to the previous question: What could be done to improve this?

- **Exploration of the effect of ADRe on health professionals' collaboration/ communication**
    - Doctors often prescribe medicines, pharmacists dispense the medicines, what, in your opinion, is the nurse's role in medicines management in the community?
    - How would you describe the effect of ADRe Profile on collaboration/communication between doctors, nurses and pharmacists? / What did you think about the way the nurses, doctors and pharmacists worked together during your ADRe medicines review?

Exploration of the effect of ADRe on patient-centred care

- Did you feel you were treated with respect? When? And how?
- Do you believe that the health professionals were concerned about your unique experience and listened to what you had to say? Please illustrate your answer with examples.
- Did you feel you were given (or were asked for) enough information about your condition/medicines/side effects? (a) Before ADRe; (b) With ADRe
- [If the response to the question above is NO]: What information was missed? Do you know how to get help or more information about your medicines or possible side effects in the future?

Interview guide for pharmacists and doctors

Interviews aim to understand:

- ADRe effectiveness (1)
- Feasibility of implementation (2)
- Views on multidisciplinary collaboration (3)

Exploration of views on ADRe Profile effectiveness

- Did you notice any patient benefit from the use of ADRe Profile?
    - What was the benefit?
    - Why/how did the benefit occur?
- In your opinion, why does ADRe Profile work/ or not work?
- Would you like to continue using the ADRe Profile? Why? How often?
- Has your view on medicines monitoring changed following your experience of using the ADRe Profile? In what way?

Exploration of the feasibility of routine implementation

- What is the usual practice with regards to medicine monitoring in your surgery? (How is it performed? How often? How long does it take? Is it performed in the same format for all patients with medicines prescriptions – and if not, what are the differences?)
- How is ADRe different from the usual practice of medicines monitoring?

- How would you describe the purpose of the ADRe Profile?/What is the point of using ADRe?/Is ADRe a good idea?
- Did the ADRe Profile promote or impede your work? In what way?
- How compatible is ADRe with the existing practices in your surgery?
- What effect did using ADRe have on monitoring the medicines use of participating patients?
- Did you encounter any problems or difficulties in utilising ADRe results
    - If YES to the previous question: What could be done to improve this?
- Was the time you spent reviewing ADRe spent usefully? /Would you be happy to review the ADRe Profile periodically for your patients?
    - If YES to the previous question: How often would you recommend that the ADRe Profile is completed for each patient?
- Can you realistically see the ADRe Profile being adopted in your practice? Are there any barriers to its implementation? (time/work pressures/lack of support from other professionals/complexity of the instrument)

- **Exploration of the effect of ADRe on the health professionals' collaboration/communication**
    - Doctors' and pharmacists' involvement with medicines is usually clearly defined within the practice, what, in your opinion, is the nurses' role in medicines management in the community (if any)?
    - How would you describe the effect of ADRe Profile on collaboration/communication between doctors, nurses and pharmacists?
    - Do you think ADRe has any impact on the division of work, resources or responsibility between different professional groups?

Interview guide for nurses/nursing assistants who implemented the ADRe Profile

Interviews aim to understand:

- ADRe effectiveness (1)
- Feasibility of implementation (2)
- Views on multidisciplinary collaboration (3)

Exploration of views on ADRe Profile effectiveness

- Did you notice any patient benefit from the use of ADRe Profile?
  - Why/how did the benefit occur?
- In your opinion, why does ADRe Profile work/ or not work?
- Would you like to continue using the ADRe Profile? Why?
- How much knowledge about medicine monitoring did you have before you started using the ADRe Profile?
- Were you involved in medicine monitoring in any way before you were involved with the study? (Is medicine monitoring part of your role?)
- Has your view on medicine monitoring changed following your experience of using the ADRe Profile? In what way?

Exploration of the feasibility of routine implementation

- What is the usual practice with regards to medicine monitoring in your surgery? (How is it performed? How often? How long does it take? Is it performed in the same format for all patients with medicines prescriptions?)
- How is ADRe different from the usual practice of medicine monitoring?
- How would you describe the purpose of the ADRe Profile? /What is the point of using ADRe?/Is ADRe a good idea?
- Did the ADRe Profile promote or impede your work? In what way?
- How compatible is ADRe with the existing practices in your surgery?
- What effect did it have on medicine monitoring of participating patients?
- Did you encounter any problems or difficulties in using the ADRe Profile?
  - If YES to the previous question: What could be done to improve this?
- Was the time you spent implementing ADRe spent usefully? /Would you be happy to use the ADRe Profile periodically for your patients?
  - If YES to the previous question: How often would you recommend that the ADRe Profile is completed for each patient?
- Can you realistically see the ADRe Profile being adopted in your practice? Are there any barriers to its implementation? (time/work pressures/lack of support from other professionals/complexity of the instrument)

- **Exploration of the effect of ADRe on the health professionals' collaboration/communication**
  - Doctors' and pharmacists' involvement with medicines is usually clearly defined within the practice, what, in your opinion, is the nurse's (or nursing assistant's) role in medicines management in the community (if any)?

- How would you describe the effect of the ADRe Profile on collaboration/communication between doctors, nurses/nursing assistants and pharmacists?
- Do you think ADRe has /may have any impact on the division of work, resources or responsibility between different professional groups?
