# Supplementary material for: Clinical Impact of Implementing a Nurse-Led Adverse Drug Reaction Profile in Older Adults Prescribed Multiple Medicines in UK Primary Care: A Study Protocol for a Cluster-Randomised Controlled Trial
Source: Pharmacy (Basel). 2022 Apr 28;10(3):52. doi: 10.3390/pharmacy10030052 (PMC9149816; doi:10.3390/pharmacy10030052)
Supplement: Supplementary file 1 [file pharmacy-10-00052-s001.zip › pharmacy-1644687-supplementary/Supplementary Table S1_Trial registration dataset, Spirit checklist, Consort checklist.pdf]

Supplementary table 1 (Table S1): Trial registration dataset, Spirit checklist, Consort checklist

Trial registration dataset (Version 1.3.1), (WHO, n.d.)

Data category	Information
Primary Registry and Trial Identifying Number	ClinicalTrials.gov<br>NCT04663360
Date of Registration in Primary Registry	26th November 2020
Secondary Identifying Numbers	Swansea University sponsor reference: RIO 034-20<br>IRAS project ID: 292693
Source(s) of Monetary or Material Support	Swansea University SURES scholarship<br>Health and Care Research Wales (HCRW), trial included on the HCRW Portfolio
Primary Sponsor	Swansea University
Secondary Sponsor(s)	Study included in the Health Care Research Wales Portfolio
Contact for Public Queries	Prof Sue Jordan: S.E.Jordan@swansea.ac.uk<br>Vera Logan: vera.logan@swansea.ac.uk
Contact for Scientific Queries	Prof Sue Jordan: S.E.Jordan@swansea.ac.uk<br>Vera Logan: vera.logan@swansea.ac.uk
Public Title	Medication monitoring in general practice: clinical impact of implementing a nurse-led Adverse Drug Reaction (ADRe) Profile in older adults with 5 or more prescribed medicines
Scientific Title	A multi-centre, pragmatic, cluster-randomised controlled trial investigating the clinical impact of implementing a nurse-led Adverse Drug Reaction (ADRe) Profile in older adults with polypharmacy in general practice
Countries of Recruitment	United Kingdom (Wales)
Health Condition(s) or Problem(s) Studied	Adverse drug reactions, polypharmacy
Intervention(s)	Intervention arm: ADRe Profile<br>Control arm: usual standard of care<br>The ADRe Profile is a nurse-led intervention designed to identify and help resolve problems and adverse side effects potentially attributable to medicines
Key Inclusion and Exclusion Criteria	Inclusion criteria:<br>• Service user, age ≥ 65 years<br>• Sex: male and female<br>• With a long-term condition<br>• Prescribed ≥ 5 medicines daily (Vitamin and nutritional supplements and moisturising skin preparations will not be counted as medicines.)<br>• Willing and able to give informed, signed consent themselves, or where capacity is lacking in the opinion of their nurses, a consultee/representative accompanying the patient

	who is willing to give advice and assent to the service user participating and sign on their behalf.<br><br>Exclusion criteria:<br>• Unable to consent and no consultee/representative present<br>• Not fluent in English or Welsh (unless a family member can assist with translation)<br>• Receiving palliative care<br>• Not expected to remain in the practice for the next 12 months
Study Type	Type of study: interventional<br>Method of allocation: cluster-randomisation<br>Masking: not used, other than for data analysis<br>Assignment: parallel<br>Purpose: effectiveness
Date of First Enrollment	October 2021
Sample Size	60
Recruitment Status	Recruiting
Primary Outcome(s)	• Calculated percentage and described nature of items on the ADRe Profile that can be populated from accessing the nursing and medical notes.<br>• Clinical impact on service users, including new problems identified (number and nature) and problems addressed (number and nature).<br>• Prescription changes (number of patients with changes in prescription regimens: drug or dose. Number and nature of changes).<br>• Description of stakeholder views on ADRe Profile implementation effectiveness (survey rating of the ADRe Profile - Likert scale).<br>• Description of stakeholder views on ADRe Profile implementation feasibility (eliciting interview themes).
Key Secondary Outcomes	• Survey of the average nurses', GP's and pharmacists' length of time per one ADRe Profile completion. Average length of the health professionals' time involvement with one ADRe Profile.<br>• Estimation of the cost of nurses', GP's and pharmacists' time, based on average national salary cost per hour.<br>• Description of the main stakeholders' views on multidisciplinary collaboration (eliciting interview themes).<br>• Description of the patients' views on the contribution of ADRe Profile to patient-centered care (eliciting interview themes).

Ethics Review	Status: approved (REC reference: 21/WA/0042)<br>IRAS project ID: 292693<br>Date of approval: 10th March 2021<br>Name and contact details of Ethics committee:<br>Wales REC 6<br>c/o Public Health Wales<br>Building 1<br>Jobswell Road<br>St David's Park<br>SA31 3HB<br>E. Wales.REC6@wales.nhs.uk<br>W. www.hra.nhs.uk

SPIRIT 2013 Checklist: Recommended items to address in a clinical trial protocol and related documents

Section/item	Item No	Description	Section No of the protocol study
**Administrative information**			
Title	1	Descriptive title identifying the study design, population, interventions, and, if applicable, trial acronym	Page 1
Trial registration	2a	Trial identifier and registry name. If not yet registered, name of intended registry	Page 1
	2b	All items from the World Health Organization Trial Registration Data Set	Appendix 1
Protocol version	3	Date and version identifier	Page 1
Funding	4	Sources and types of financial, material, and other support	9
Roles and responsibilities	5a	Names, affiliations, and roles of protocol contributors	10
	5b	Name and contact information for the trial sponsor	3.12

	5c	Role of study sponsor and funders, if any, in study design; collection, management, analysis, and interpretation of data; writing of the report; and the decision to submit the report for publication, including whether they will have ultimate authority over any of these activities	3.12
	5d	Composition, roles, and responsibilities of the coordinating centre, steering committee, endpoint adjudication committee, data management team, and other individuals or groups overseeing the trial, if applicable (see Item 21a for data monitoring committee)	3.10
**Introduction**			
Background and rationale	6a	Description of research question and justification for undertaking the trial, including summary of relevant studies (published and unpublished) examining benefits and harms for each intervention	1
	6b	Explanation for choice of comparators	3.2
Objectives	7	Specific objectives or hypotheses	1
Trial design	8	Description of trial design including type of trial (eg, parallel group, crossover, factorial, single group), allocation ratio, and framework (eg, superiority, equivalence, noninferiority, exploratory)	3
**Methods: Participants, interventions, and outcomes**			
Study setting	9	Description of study settings (eg, community clinic, academic hospital) and list of countries where data will be collected. Reference to where list of study sites can be obtained	3.1
Eligibility criteria	10	Inclusion and exclusion criteria for participants. If applicable, eligibility criteria for study centres and individuals who will perform the interventions (eg, surgeons, psychotherapists)	3.1
Interventions	11a	Interventions for each group with sufficient detail to allow replication, including how and when they will be administered	3.2
	11b	Criteria for discontinuing or modifying allocated interventions for a given trial participant (eg, drug dose change in response to harms, participant request, or improving/worsening disease)	3.11

	11c	Strategies to improve adherence to intervention protocols, and any procedures for monitoring adherence (eg, drug tablet return, laboratory tests)	N/A
	11d	Relevant concomitant care and interventions that are permitted or prohibited during the trial	3.2
Outcomes	12	Primary, secondary, and other outcomes, including the specific measurement variable (eg, systolic blood pressure), analysis metric (eg, change from baseline, final value, time to event), method of aggregation (eg, median, proportion), and time point for each outcome. Explanation of the clinical relevance of chosen efficacy and harm outcomes is strongly recommended	3.3
Participant timeline	13	Time schedule of enrolment, interventions (including any run-ins and washouts), assessments, and visits for participants. A schematic diagram is highly recommended (see Figure)	3.7 (table 1)
Sample size	14	Estimated number of participants needed to achieve study objectives and how it was determined, including clinical and statistical assumptions supporting any sample size calculations	3.5
Recruitment	15	Strategies for achieving adequate participant enrolment to reach target sample size	3.1
**Methods: Assignment of interventions (for controlled trials)**			
Allocation:			
Sequence generation	16a	Method of generating the allocation sequence (eg, computer-generated random numbers), and list of any factors for stratification. To reduce predictability of a random sequence, details of any planned restriction (eg, blocking) should be provided in a separate document that is unavailable to those who enrol participants or assign interventions	3.6
Allocation concealment mechanism	16b	Mechanism of implementing the allocation sequence (eg, central telephone; sequentially numbered, opaque, sealed envelopes), describing any steps to conceal the sequence until interventions are assigned	N/A
Implementation	16c	Who will generate the allocation sequence, who will enrol participants, and who will assign participants to interventions	3.6, 3.7

Blinding (masking)	17a	Who will be blinded after assignment to interventions (eg, trial participants, care providers, outcome assessors, data analysts), and how	N/A
	17b	If blinded, circumstances under which unblinding is permissible, and procedure for revealing a participant's allocated intervention during the trial	N/A
**Methods: Data collection, management, and analysis**			
Data collection methods	18a	Plans for assessment and collection of outcome, baseline, and other trial data, including any related processes to promote data quality (eg, duplicate measurements, training of assessors) and a description of study instruments (eg, questionnaires, laboratory tests) along with their reliability and validity, if known. Reference to where data collection forms can be found, if not in the protocol	3.7
	18b	Plans to promote participant retention and complete follow-up, including list of any outcome data to be collected for participants who discontinue or deviate from intervention protocols	3.7
Data management	19	Plans for data entry, coding, security, and storage, including any related processes to promote data quality (eg, double data entry; range checks for data values). Reference to where details of data management procedures can be found, if not in the protocol	3.8, 5.2
Statistical methods	20a	Statistical methods for analysing primary and secondary outcomes. Reference to where other details of the statistical analysis plan can be found, if not in the protocol	3.9
	20b	Methods for any additional analyses (eg, subgroup and adjusted analyses)	3.9
	20c	Definition of analysis population relating to protocol non-adherence (eg, as randomised analysis), and any statistical methods to handle missing data (eg, multiple imputation)	N/A
**Methods: Monitoring**			

Data monitoring	21a	Composition of data monitoring committee (DMC); summary of its role and reporting structure; statement of whether it is independent from the sponsor and competing interests; and reference to where further details about its charter can be found, if not in the protocol. Alternatively, an explanation of why a DMC is not needed	3.10
	21b	Description of any interim analyses and stopping guidelines, including who will have access to these interim results and make the final decision to terminate the trial	3.11
Harms	22	Plans for collecting, assessing, reporting, and managing solicited and spontaneously reported adverse events and other unintended effects of trial interventions or trial conduct	3.11
Auditing	23	Frequency and procedures for auditing trial conduct, if any, and whether the process will be independent from investigators and the sponsor	N/A
**Ethics and dissemination**			
Research ethics approval	24	Plans for seeking research ethics committee/institutional review board (REC/IRB) approval	5
Protocol amendments	25	Plans for communicating important protocol modifications (eg, changes to eligibility criteria, outcomes, analyses) to relevant parties (eg, investigators, REC/IRBs, trial participants, trial registries, journals, regulators)	5
Consent or assent	26a	Who will obtain informed consent or assent from potential trial participants or authorised surrogates, and how (see Item 32)	5.1
	26b	Additional consent provisions for collection and use of participant data and biological specimens in ancillary studies, if applicable	N/A
Confidentiality	27	How personal information about potential and enrolled participants will be collected, shared, and maintained in order to protect confidentiality before, during, and after the trial	5.2
Declaration of interests	28	Financial and other competing interests for principal investigators for the overall trial and each study site	9

Access to data	29	Statement of who will have access to the final trial dataset, and disclosure of contractual agreements that limit such access for investigators	8
Ancillary and post-trial care	30	Provisions, if any, for ancillary and post-trial care, and for compensation to those who suffer harm from trial participation	3.13
Dissemination policy	31a	Plans for investigators and sponsor to communicate trial results to participants, healthcare professionals, the public, and other relevant groups (eg, via publication, reporting in results databases, or other data sharing arrangements), including any publication restrictions	5.3
	31b	Authorship eligibility guidelines and any intended use of professional writers	10
	31c	Plans, if any, for granting public access to the full protocol, participant-level dataset, and statistical code	5.3, 8
**Appendices**			
Informed consent materials	32	Model consent form and other related documentation given to participants and authorised surrogates	Appendix 4
Biological specimens	33	Plans for collection, laboratory evaluation, and storage of biological specimens for genetic or molecular analysis in the current trial and for future use in ancillary studies, if applicable	N/A

CONSORT 2010 checklist of information to include when reporting a cluster randomised trial

Section/Topic	Item No	Standard Checklist item	Extension for cluster designs	Page No *
**Title and abstract**				
	1a	Identification as a randomised trial in the title	Identification as a cluster randomised trial in the title	1
	1b	Structured summary of trial design, methods, results, and conclusions (for specific	See table 2	1

		guidance see CONSORT for abstracts)[i,ii]		
**Introduction**				
**Background and objectives**	2a	Scientific background and explanation of rationale	Rationale for using a cluster design	Section 1
	2b	Specific objectives or hypotheses	Whether objectives pertain to the the cluster level, the individual participant level or both	Section 1
**Methods**				
**Trial design**	3a	Description of trial design (such as parallel, factorial) including allocation ratio	Definition of cluster and description of how the design features apply to the clusters	Section 3.1
	3b	Important changes to methods after trial commencement (such as eligibility criteria), with reasons		**N/A**
**Participants**	4a	Eligibility criteria for participants	Eligibility criteria for clusters	Section 3.1
	4b	Settings and locations where the data were collected		**Section 3.1**
**Interventions**	5	The interventions for each group with sufficient details to allow replication, including how and when they were actually administered	Whether interventions pertain to the cluster level, the individual participant level or both	Section 3.2
**Outcomes**	6a	Completely defined pre-specified primary and secondary outcome measures, including how and when they were assessed	Whether outcome measures pertain to the cluster level, the individual participant level or both	Section 3.3
	6b	Any changes to trial outcomes after the trial commenced, with reasons		**N/A**
**Sample size**	7a	How sample size was determined	Method of calculation, number of clusters(s) (and whether equal or unequal cluster sizes are assumed), cluster size, a coefficient of intracluster correlation (ICC or k), and an indication of its uncertainty	Section 3.5
	7b	When applicable, explanation of any interim analyses and stopping guidelines		**Section 3.11**

**Randomisation:**				
**Sequence generation**	8a	Method used to generate the random allocation sequence		**Section 3.6**
	8b	Type of randomisation; details of any restriction (such as blocking and block size)	Details of stratification or matching if used	Section 3.6
**Allocation concealment mechanism**	9	Mechanism used to implement the random allocation sequence (such as sequentially numbered containers), describing any steps taken to conceal the sequence until interventions were assigned	Specification that allocation was based on clusters rather than individuals and whether allocation concealment (if any) was at the cluster level, the individual participant level or both	Section 3.6
**Implementation**	10	Who generated the random allocation sequence, who enrolled participants, and who assigned participants to interventions	Replace by 10a, 10b and 10c	Section 3.7
	10a		Who generated the random allocation sequence, who enrolled clusters, and who assigned clusters to interventions	Section 3.7
	10b		Mechanism by which individual participants were included in clusters for the purposes of the trial (such as complete enumeration, random sampling)	Section 3.6
	10c		From whom consent was sought (representatives of the cluster, or individual cluster members, or both), and whether consent was sought before or after randomisation	3.7
**Blinding**	11a	If done, who was blinded after assignment to interventions (for example, participants, care providers, those assessing outcomes) and how		**N/A**
	11b	If relevant, description of the similarity of interventions		**N/A**

**Statistical methods**	12a	Statistical methods used to compare groups for primary and secondary outcomes	How clustering was taken into account	Section 3.9
	12b	Methods for additional analyses, such as subgroup analyses and adjusted analyses		**Section 3.9**
**Results**				
**Participant flow (a diagram is strongly recommended)**	13a	For each group, the numbers of participants who were randomly assigned, received intended treatment, and were analysed for the primary outcome	For each group, the numbers of clusters that were randomly assigned, received intended treatment, and were analysed for the primary outcome	Section 3.7
	13b	For each group, losses and exclusions after randomisation, together with reasons	For each group, losses and exclusions for both clusters and individual cluster members	N/A
**Recruitment**	14a	Dates defining the periods of recruitment and follow-up		**Section 3.7 (table 1)**
	14b	Why the trial ended or was stopped		**N/A**
**Baseline data**	15	A table showing baseline demographic and clinical characteristics for each group	Baseline characteristics for the individual and cluster levels as applicable for each group	N/A
**Numbers analysed**	16	For each group, number of participants (denominator) included in each analysis and whether the analysis was by original assigned groups	For each group, number of clusters included in each analysis	N/A
**Outcomes and estimation**	17a	For each primary and secondary outcome, results for each group, and the estimated effect size and its precision (such as 95% confidence interval)	Results at the individual or cluster level as applicable and a coefficient of intracluster correlation (ICC or k) for each primary outcome	N/A
	17b	For binary outcomes, presentation of both absolute and relative effect sizes is recommended		**N/A**
**Ancillary analyses**	18	Results of any other analyses performed, including subgroup analyses and adjusted analyses,		**N/A**

		distinguishing pre-specified from exploratory		
**Harms**	19	All important harms or unintended effects in each group (for specific guidance see CONSORT for harms[iii])		**N/A**
**Discussion**				
**Limitations**	20	Trial limitations, addressing sources of potential bias, imprecision, and, if relevant, multiplicity of analyses		**Section 6**
**Generalisability**	21	Generalisability (external validity, applicability) of the trial findings	Generalisability to clusters and/or individual participants (as relevant)	Section 6
**Interpretation**	22	Interpretation consistent with results, balancing benefits and harms, and considering other relevant evidence		**N/A**
**Other information**				
**Registration**	23	Registration number and name of trial registry		**Page 1**
**Protocol**	24	Where the full trial protocol can be accessed, if available		**Corresponding author**
**Funding**	25	Sources of funding and other support (such as supply of drugs), role of funders		**Section 9**

** Note: page numbers optional depending on journal requirements*

Extension of CONSORT for abstracts[iii] to reports of cluster randomised trials

Item	Standard Checklist item	Extension for cluster trials	Included
**Title**	Identification of study as randomised	**Identification of study as cluster randomised**	yes
**Trial design**	Description of the trial design (e.g. parallel, cluster, non-inferiority)		yes
**Methods**			
**Participants**	Eligibility criteria for participants and the settings where the data were collected	**Eligibility criteria for clusters**	yes
**Interventions**	Interventions intended for each group		yes
**Objective**	Specific objective or hypothesis	**Whether objective or hypothesis pertains to the cluster level, the individual participant level or both**	yes
**Outcome**	Clearly defined primary outcome for this report	**Whether the primary outcome pertains to the cluster level, the individual participant level or both**	yes
**Randomization**	How participants were allocated to interventions	**How clusters were allocated to interventions**	yes
**Blinding (masking)**	Whether or not participants, care givers, and those assessing the outcomes were blinded to group assignment		N/A
**Results**			
**Numbers randomized**	Number of participants randomized to each group	**Number of clusters randomized to each group**	N/A
**Recruitment**	Trial status[1]		
**Numbers analysed**	Number of participants analysed in each group	**Number of clusters analysed in each group**	N/A
**Outcome**	For the primary outcome, a result for each group and the estimated effect size and its precision	**Results at the cluster or individual participant level as applicable for each primary outcome**	N/A
**Harms**	Important adverse events or side effects		N/A
**Conclusions**	General interpretation of the results		N/A

[1] Relevant to Conference Abstracts

**Trial registration**	Registration number and name of trial register
**Funding**	Source of funding

REFERENCES

i Hopewell S, Clarke M, Moher D, Wager E, Middleton P, Altman DG, et al. CONSORT for reporting randomised trials in journal and conference abstracts. *Lancet* 2008, 371:281-283

ii Hopewell S, Clarke M, Moher D, Wager E, Middleton P, Altman DG at al (2008) CONSORT for reporting randomized controlled trials in journal and conference abstracts: explanation and elaboration. *PLoS Med* 5(1): e20

iii Ioannidis JP, Evans SJ, Gotzsche PC, O'Neill RT, Altman DG, Schulz K, Moher D. Better reporting of harms in randomized trials: an extension of the CONSORT statement. *Ann Intern Med* 2004; 141(10):781-788.
