# Supplementary material for: Clinical Impact of Implementing a Nurse-Led Adverse Drug Reaction Profile in Older Adults Prescribed Multiple Medicines in UK Primary Care: A Study Protocol for a Cluster-Randomised Controlled Trial
Source: Pharmacy (Basel). 2022 Apr 28;10(3):52. doi: 10.3390/pharmacy10030052 (PMC9149816; doi:10.3390/pharmacy10030052)
Supplement: Supplementary file 1 [file pharmacy-10-00052-s001.zip › pharmacy-1644687-supplementary/Supplementary Table S2_Fidelity measurement.pdf]

Supplementary table 2 (Table S2): Fidelity measurement, core components and permitted adaptations [based on a modified version of the Conceptual Framework for Implementation Fidelity (Carroll et al., 2007; Hasson, 2010)]

	Descriptors of fidelity	Descriptors of local adaptation<br>To be completed after all data are collected
Content	Medical notes accessed before randomisation<br>ADRe questions completed<br>- Vital signs (__/10)<br>- Observations (__/18)<br>- Reports (__/41)<br>- Medicines administration (__/6)<br>- Health promotion (__/14)<br>Health promotion feedback provided<br>ADR report escalated to a prescriber<br>Action to resolve identified problems	
Delivery	ADRe Profile completed by the researcher/practice nurse/Health care support worker?<br>Completion in GP practice/service user home?	
Frequency	ADRe Profile administered twice?	
Duration	Duration of completion between 10 min and 1 hr?	
Coverage	Number of participants who had ADRe Profile administered (n=30)	
Timeliness	Time interval between 2 ADRe Profile completions: more than 1 month, less than 3 months	

Implementation moderators:

<u>Quality of delivery:</u>

Was training provided for prescribers and persons delivering the ADRe Profile intervention?

Were instructions for self-completion provided to service users?

Confidentiality of personal and medical data respected?

Description of stakeholder views on multidisciplinary collaboration

Description of patient views on the contribution of ADRe Profile to patient-centred care

<u>Participant responsiveness:</u>

Qualitative insights from the field diary
