# Supplementary material for: Clinical Impact of Implementing a Nurse-Led Adverse Drug Reaction Profile in Older Adults Prescribed Multiple Medicines in UK Primary Care: A Study Protocol for a Cluster-Randomised Controlled Trial
Source: Pharmacy (Basel). 2022 Apr 28;10(3):52. doi: 10.3390/pharmacy10030052 (PMC9149816; doi:10.3390/pharmacy10030052)
Supplement: Supplementary file 1 [file pharmacy-10-00052-s001.zip › pharmacy-1644687-supplementary/Supplementary Table S3_GANTT chart of research activities.pdf]

Supplementary table 3 (Table S3): GANTT chart of research activities (D – data collection, I – intervention, R – randomisation)

Time-frame:		Month 1 + 2+3		**Round 1** Month 3 + 4 + 5 + 6				**Round 2** Month 9 + 10 + 11 + 12				Month 13 - 18
**Specific Activity**	**Undertaken by**	**Screen**	**Enrol**	**D1**	**R**	**I1**	**Review**	**D2**	**I2**	**Review**		**Interviews**
Selection and screening	Study Pharmacist	x										
Informed consent	Practice nurse/Researcher		X									
Medical history, problems and prescriptions from clinical records	Researchers			x				x				
Randomisation	Statistician				x							
Instructions/education for patient and/or care giver	Researcher			x				x				
Self-completion of the ADRe Profile	Patient					x			x			
Administration of the ADRe Profile	Practice nurse/Researcher					x			x			
Pharmacist review of the ADRe Profile and list of medicines/MAR chart	Pharmacist						x			X		
GP review of pharmacist recommendations	GP						x			X		
Dissemination of study results to participants	Researcher										X	
Participant survey	Researcher									X		
Stakeholder interviews	Researcher											X
